# Neurological, cardiac, musculoskeletal, and renal manifestations of scleroderma along with insights into its genetics, pathophysiology, diagnostic, and therapeutic updates

**DOI:** 10.1002/hsr2.2072

**Published:** 2024-04-24

**Authors:** Priyadarshi Prajjwal, Mohammed Dheyaa Marsool Marsool, Vikas Yadav, Ramya S. D. Kanagala, Yeruva Bheemeswara Reddy, Jobby John, Justin Riley Lam, Nanditha Karra, Bita Amiri, Moiz Ul Islam, Venkatesh Nithya, Ali Dheyaa Marsool Marsool, Srikanth Gadam, Neel Vora, Omniat Amir Hussin

**Affiliations:** ^1^ Department of Neurology Bharati Vidyapeeth Deemed University Pune India; ^2^ Department of Neurology, Al‐Kindy College of Medicine University of Baghdad Baghdad Iraq; ^3^ Department of Internal Medicine Pt. B. D. S. Postgraduate Institute of Medical Sciences Rohtak India; ^4^ Department of Internal Medicine Mamata Medical College Khammam Telangana India; ^5^ Department of Internal Medicine Zaporozhyea State Medical University Zaporozhyea Ukraine; ^6^ Department of Internal Medicine Dr. Somervell Memorial CSI Medical College and Hospital Neyyāttinkara India; ^7^ Department of Internal Medicine Cebu Institute of Medicine Cebu Philippines; ^8^ Department of Internal Medicine Osmania Medical College Hyderabad Telangana India; ^9^ Cardiovascular Research Center Tabriz University of Medical Sciences Tabriz Iran; ^10^ Department of Internal Medicine Punjab Medical College Faisalabad Pakistan; ^11^ Department of Internal Medicine S. D. Asfendiyarov Kazakh National Medical University Almaty Kazakhstan; ^12^ Mayo Clinic Rochester Minnesota USA; ^13^ B. J. Medical College Ahmedabad India; ^14^ Department of Medicine Almanhal University Academy of Science Khartoum Sudan

**Keywords:** cardiac abnormalities, genetics, neurological manifestations, renal complications, scleroderma, systemic sclerosis, therapeutic updates

## Abstract

**Background:**

Scleroderma, also referred to as systemic sclerosis, is a multifaceted autoimmune condition characterized by abnormal fibrosis and impaired vascular function. Pathologically, it encompasses the persistent presence of inflammation, abnormal collagen buildup, and restructuring of blood vessels in various organs, resulting in a wide range of clinical symptoms. This review incorporates the most recent scientific literature on scleroderma, with a particular emphasis on its pathophysiology, clinical manifestations, diagnostic approaches, and treatment options.

**Methodology:**

A comprehensive investigation was carried out on numerous databases, such as PubMed, MEDLINE, Scopus, Web of Science, and Google Scholar, to collect pertinent studies covering diverse facets of scleroderma research.

**Results:**

Scleroderma presents with a range of systemic manifestations, such as interstitial lung disease, gastrointestinal dysmotility, Raynaud's phenomenon, pulmonary arterial hypertension, renal complications, neurological symptoms, and cardiac abnormalities. Serological markers, such as antinuclear antibodies, anti‐centromere antibodies, and anti‐topoisomerase antibodies, are important for classifying diseases and predicting their outcomes.

**Discussion:**

The precise identification of scleroderma is crucial for promptly and correctly implementing effective treatment plans. Treatment approaches aim to improve symptoms, reduce complications, and slow down the progression of the disease. An integrated approach that combines pharmacological agents, including immunosuppressants, endothelin receptor antagonists, and prostanoids, with nonpharmacological interventions such as physical and occupational therapy is essential for maximizing patient care.

**Conclusion:**

Through the clarification of existing gaps in knowledge and identification of emerging trends, our goal is to improve the accuracy of diagnosis, enhance the effectiveness of therapeutic interventions, and ultimately enhance the overall quality of life for individuals suffering from scleroderma. Ongoing cooperation and creative research are necessary to advance the field and achieve improved patient outcomes and new therapeutic discoveries.

## 1 BACKGROUND

Scleroderma, also referred to as systemic sclerosis (SSc), is a complex autoimmune condition that involves various pathological changes, including excessive fibrosis and vascular abnormalities.^1^, ^2^ The pathological changes are caused by an immune response that leads to long‐lasting inflammation, resulting in excessive production of collagen and other components of the extracellular matrix (ECM).^3^ This dysregulated fibrosis extends its impact to various tissues and organs, including the skin, lungs, gastrointestinal tract, heart, and kidneys. Concurrently, vascular dysfunction, marked by vascular remodeling and endothelial cell injury, further exacerbates the disease process, contributing to its complexity.^4^, ^5^, ^6^

From a clinical perspective, scleroderma presents itself in a wide range of ways. Remarkably, a distinguishing characteristic of this condition is the impact it has on the skin, characterized by thickening, hardening, and tightness.^7^ These symptoms have a profound effect on both physical and psychological health. In addition to skin manifestations, scleroderma also involves systemic features like Raynaud's phenomenon.^7^, ^8^ This vascular disorder is characterized by abnormal spasms in response to cold or stress, leading to color changes and pain in the extremities.^9^, ^10^ In addition, patients may also experience gastrointestinal dysmotility, interstitial lung disease (ILD), pulmonary arterial hypertension (PAH), renal complications, and cardiac abnormalities, which can further complicate the clinical picture.^1^, ^9^

Understanding the pathogenesis, clinical presentation, and diagnostic approach to scleroderma is imperative for effective management and therapeutic intervention. The disease's pathogenesis involves intricate interplay among genetic predisposition, environmental triggers, and dysregulated immune responses.^1^, ^3^ Genetic studies have identified susceptibility loci, including HLA alleles and genes implicated in immune regulation and ECM remodeling.^3^ Clinically, the diagnosis of scleroderma relies on a combination of clinical features, serological markers, and imaging studies. Serological markers such as anti‐nuclear antibodies (ANA), anti‐topoisomerase I (Scl‐70), and anti‐centromere antibodies (ACA) aid in disease classification and prognostication, while imaging modalities such as high‐resolution computed tomography and echocardiography facilitate the assessment of organ involvement and disease progression.^1^, ^2^, ^3^

This review aims to make a valuable contribution to the scientific community's ongoing efforts to improve patient care, enhance diagnostic accuracy, and develop effective treatments for individuals affected by scleroderma. Through a deep understanding of the complex mechanisms involved in the disease and the exploration of cutting‐edge therapeutic approaches, our goal is to reduce the impact of scleroderma and enhance the well‐being of those affected.

## 2 METHODOLOGY

### 2.1 Literature search

We performed an extensive search to conduct a comprehensive narrative review on Scleroderma and its various aspects. Databases such as PubMed, MEDLINE, Scopus, Web of Science, and Google Scholar were utilized to identify relevant scientific papers, research articles, reviews, clinical trials, and case reports published up to the current date. The search terms used were “Scleroderma,” AND/OR “Systemic sclerosis,” AND “Autoimmune disease,” AND/OR, “Genetics,” AND, “Neurological manifestations,” AND/OR, “Cardiac abnormalities,” AND/OR “Musculoskeletal involvement,” AND/OR “Renal complications,” AND “Pathophysiology,” “Treatment of Scleroderma,” and finally, “Therapeutic updates.” A well‐designed search strategy was created to methodically search the chosen databases. A variety of search terms and Boolean operators (AND/OR) were used to gather a wide range of literature that is pertinent to the objectives of the review. The search focused on topics such as the genetic basis, neurological and cardiac involvement, musculoskeletal issues, renal complications, the underlying pathophysiology, and the latest advancements in treatment options for scleroderma.

Titles and abstracts were carefully reviewed to ensure they aligned with the objectives of the review. This was followed by a comprehensive analysis of the full‐text articles to determine if they met the criteria for inclusion. Excluded from the review were publications and articles that were duplicates or did not meet the inclusion criteria.

### 2.2 Inclusion and exclusion criteria

Criteria were established to guide the selection of literature for review. We focused on peer‐reviewed articles published in English. To offer a thorough understanding of the existing literature. The inclusion criteria were as follows:

- Relevance to the topic and content related to neurological, cardiac, musculoskeletal, and renal manifestations of scleroderma, its genetics, pathophysiology, diagnosis, and therapeutic updates.
- Only peer‐reviewed, English‐language articles were considered.
- Studies published before the knowledge cutoff date of September 2022 were included.
- Studies with full text access were only included.

We excluded studies that:

- Were not relevant to our topic of scleroderma.
- Were duplicates or unavailable in full‐text format.
- Were letters, editorials, or conference abstracts.

## 3 RESULTS

Our review provided insights into the genetics, pathophysiology, and multisystemic involvement of scleroderma. The most frequently reported genes were human leukocyte antigens (HLA), specifically HLA‐DRB1 and HLA‐DQB1, STAT4, IRF5, and TGF‐β. Our study revealed a multifaceted interplay of immune dysregulation, endothelial dysfunction, and abnormal tissue fibrosis in scleroderma. These processes contribute to tissue damage, vascular complications, and progressive fibrosis, which are characteristic features of the disease. The most common clinical features included skin involvement, such as sclerodactyly and digital ulcers (DUs), and multisystem involvement, especially in the lungs, GIT, renal, CNS, and CVS. Pharmacological treatment included immunosuppressive agents, such as methotrexate and mycophenolate mofetil, for suppressing autoimmune‐mediated inflammation and fibrosis. Vasodilators, such as calcium channel blockers and phosphodiesterase‐5 inhibitors, were prescribed to alleviate vascular symptoms and improve digital blood flow. Additionally, targeted biologic therapies, including anti‐fibrotic agents and monoclonal antibodies against specific cytokines, held promise for modulating key pathogenic pathways in scleroderma. However, the heterogeneity of scleroderma manifestations and variable treatment responses underscored the need for personalized therapeutic approaches tailored to individual patient characteristics and disease subsets.

## 4 DISCUSSION

### 4.1 Pathophysiology of scleroderma

**Figure 1:**
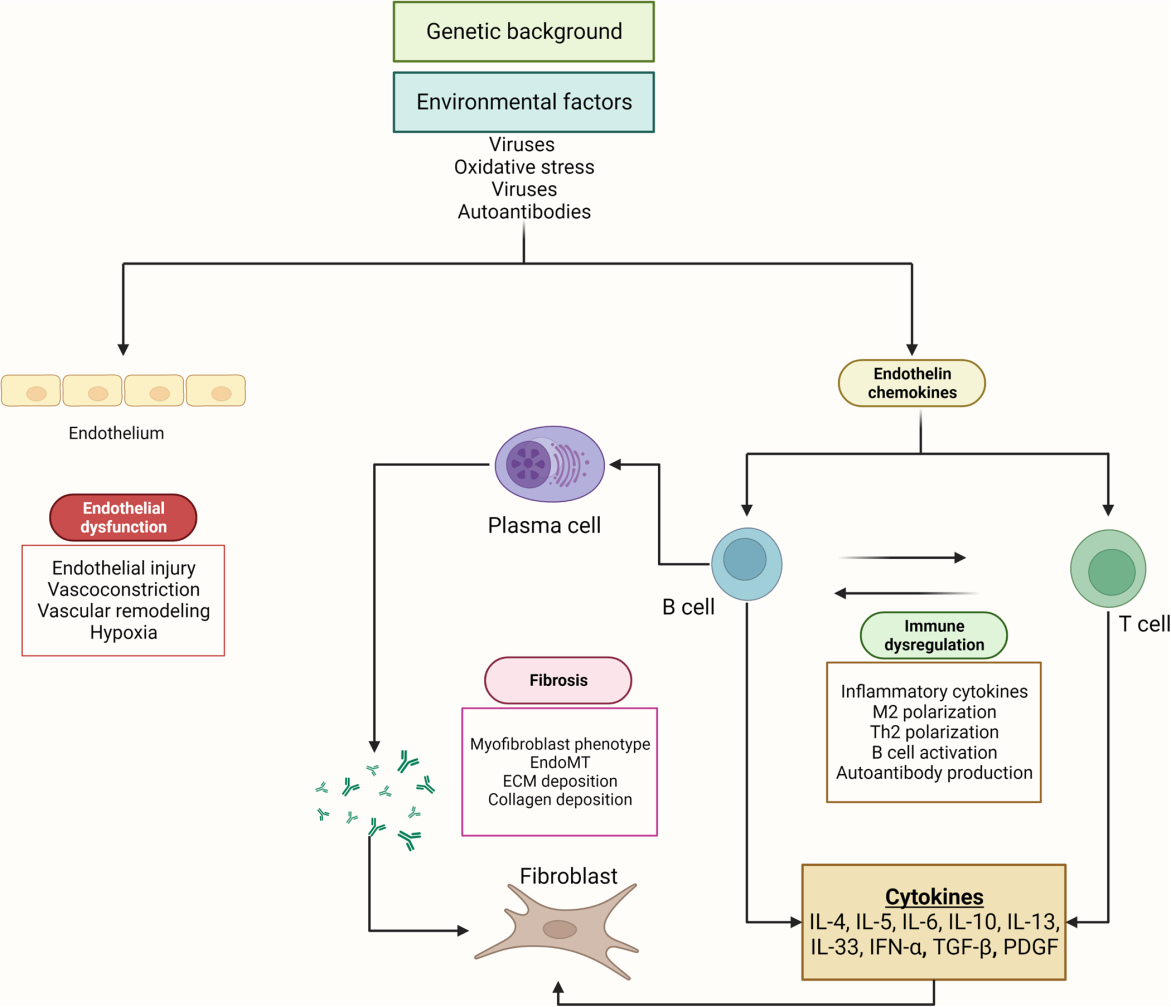
Simplified scheme showing the main processes involved in the pathogenesis of systemic sclerosis (original figure, made with Biorender).

The exact mechanism for developing scleroderma still needs to be elucidated. However, multiple mechanisms have been demonstrated to affect the development of this disease. And the different types of scleroderma shares some of the same features (Figure 1). One significant factor contributing to scleroderma is the overshooting of the immune response in the vascular tissue.^2^ There is also evidence that autoimmunity with specific autoantibodies and invigoration of innate and adaptive immunity leads to irreversible scarring and organ due to the skin's and visceral organs' fibrosis.^3^ Genetics has also been shown to play a part in the pathogenesis of this disease. Various genes have been identified; however, none would be the definitive cause of the disease.^4^ Environmental factors may also have a role; studies have shown that silica dust and certain medications may initiate the disease process; some examples include silica, toluene, and xylene. Infectious agents, including Cytomegalovirus, Epstein virus, and Parvovirus, have been associated with this condition.^5^

The pathogenesis of scleroderma involves fibrosis, tissue damage, and vascular dysfunction as well as multiple fibrotic cytokines and chemokines, alongside injury by reactive oxygen species. The imbalance of these factors through their interplay leads to such changes.^7^ Scleroderma also increases collagen accumulation in the skin, specifically the dermis. This occurs above all due to fibroblast activation and exorbitant synthesis of collagen and other matrix proteins. This also leads to the imbalance of anabolic and catabolic processes in the skin, with the excess output passing the metalloproteinase's ability to degrade the proteins.^8^ Immune cells also play a significant role in secreting several cytokines that increase organ fibrosis formation. These include transforming growth factor (TGF)‐β, interleukin (IL‐4), and IL‐13. These cytokines are said to be “fibrogenic” and significantly account for the clinical signs and symptoms of scleroderma.^9^ Scleroderma patients are shown to have a characteristic excess of these “fibrogenic” factors in their serum.^10^ The ability of these factors to induce fibrosis can be seen in different models, where it is exhibited that the absence or inhibition of these “fibrogenic” factors leads to the prevention of the fibrosis found characteristically in scleroderma.^11^ Type 2 helper (Th2) cells mostly secrete the “fibrogenic” factors IL‐4 and IL‐13. Both factors can induce an immune response that stimulates B cell proliferation and synthesis of immunoglobulin and adhesion molecules, contributing to auto‐antibodies formation in scleroderma.^12^

Auto‐antibodies detected in scleroderma are one of the most common manifestations, observed in almost all patients; the ACA are the most prevalent lab findings for limited scleroderma; this is reported in half of the patients compared with only 10% of patients with diffuse Scleroderma, and may correlate with PAH due to the vascular remodeling.^13^ The presence of the antibodies helps diagnose patients with scleroderma, which can even differentiate diffuse from local scleroderma. Its value in the pathogenesis of the condition is still vague given that some patients with diffuse scleroderma, who have both the anti‐topoisomerase antibodies (ATA) and antibodies, are no more severe than patients with diffuse who have only ATA.^14^ This suggests that detecting these auto‐antibodies has specific predictive and diagnostic use; its role in the pathophysiology may not be as important as once thought. Another “fibrogenic” cytokine is the platelet‐derived growth factor (PDGF), a receptor part of a large family of complex proteins. PDGF itself may be formed of four different protein chains composed of pairs that can assemble as either homodimers or heterodimers.

Additionally, there are two types of receptor chains: α and β, which function as dimers. For PDGF to activate, the proper conformation of both chains, through its combination, allows the latent tyrosine kinase activity to be activated.^14^ In general, the activation of PDGF and its intrinsic tyrosine kinase signals goes through Ras to MAP kinase pathways and may impel NAPDH oxidases and factors that activate transcription, which increases the formation of ECM components like collagen, which leads to the characteristic fibrosis seen in scleroderma.^15^ The activation of these factors leads to the stimulation of fibroblasts. These fibroblasts are derived from the subcutaneous layer of the skin and are the effector cells for the transcription factors.^16^

The fibroblasts can form the ECM and have myofibroblast characteristics; this allows the fibroblast to create collagen and contract to develop the mechanical stability for wound repair. The fibrosis forms a stiff, collagen‐rich scar with stability below the strength before the formation of fibrosis. Myofibroblasts in systemic scleroderma are constantly activated by the activation factors, leading to the uncontrolled deposition and production of ECM proteins. The scarring prevents the organ from functioning correctly, resulting in failure.^17^ Again, the combination of cellular and molecular changes is poorly understood. Still, it seems that the processes that interact with each other come from the different immune cells, such as mast cells, dendritic cells, and T and B lymphocytes. The synthesis and secretion of various cytokines, growth factors, interferons, autoantibodies, and enzymes all play a minor part in the complex pathophysiology of this disease. Genetics may also have an impact on the development of this disease. Although no one gene is labeled as the cause of scleroderma, multiple genes have been noted to be associated with the condition. Numerous loci are present in patients with scleroderma; this includes various HLA‐D loci, PTPN22, and IRF5.^18^, ^19^

### 4.2 Musculoskeletal involvement in scleroderma

The basic notion of SSc is immune activation, vasculopathy, and excessive and widespread fibrosis.^20^ During the inflammatory phase, a molecular mimicry occurs between infection and autoimmune antibodies, which is not lucid.^21^ However, few recent studies found ANA target specific antigens linked. Depending on characteristics associations, each type has a particular target antigen like topoisomerase‐I of diffuse cutaneous SSc (dcSSc) causes tendon friction rubs (TFRs), centromere proteins of limited cutaneous SSc (lcSSc) cause digital ischemic ulcers, anti‐RNA polymerase III of dcSSc causes both TFRs & joint contractures. Female to male ratio is 3:1, and 50% of patients would have <40 years age of onset.^22^, ^23^ Meanwhile, myositis has numerous target antigens, such as U3‐RNP (fibrillarin), Pm/scl, ku, and U1‐RNP.^23^, ^24^, ^25^ The various types of musculoskeletal involvement are articular, tendon, bone, soft tissue, and peripheral nervous system. In particular, it is classified further into articular and non‐articular, as the former type leads to arthralgia, inflammatory arthritis, psoriatic arthritis, osteogenic/degeneration, and erosive or non‐erosive arthritis. At the same time, the latter induces shortening or loss of digits.^24^, ^25^, ^26^, ^27^, ^28^, ^29^

#### 4.2.1 Articular involvement

Though controversies are present, it is widely accepted and believed that arthritis is the foremost symptom to causes paramount irrevocable changes in SSc patients, limiting their daily activities.^30^ It has manifold forms of clinical presentations, can be acute or intermittent, chronic or subacute, and slowly or rapidly progressive by involving mono, oligo, or polyarticular pattern.^31^ Based on widely accepted European Scleroderma Trials and Research (EUSTAR) cohort studies, it is figured that the prevalence of joint contractures, TFR, and synovitis were 31%, 11%, and 16%, respectively.^32^ Nevertheless, most Polyarthralgia and joint contractures are primarily associated with dcSSc. In contrast, stiffness and mild arthritis are noticed in lcSSc of small and large joints, another difference is the severity, and early presentation is most frequent in dcSSc compared to limited.^33^

In recent studies, arthritis was found more in metacarpophalangeal joints (MCP), wrists, knees, distal interphalangeal joints (DIP), and proximal interphalangeal joints (PIP) in declining pattern.^34^ Few meta‐analyses of recent studies suggested overlap of rheumatoid arthritis (RA) and scleroderma is possible as they both endanger normal human joints^35^; adding further, it's been arduous for the precise conclusion of their linkage for the reason that similar pattern of joint destruction is seen in both disorders.^34^, ^35^, ^36^ However, a genetic study identified Human Leukocyte Antigen death receptor 3 (HLA‐DR 3) and HLA‐DR 1 alleles of SSc, HLA‐DR 1, and HLA‐DR 4 of RA have some overlaps in SSc‐RA patients.^37^ Furthermore, anti‐CCP antibodies and rheumatoid factor (RF) is seen in SSc patients; nonetheless, their presence does not support RA in SSc even in the presence of anti‐a galactosyl IgG antibodies due to insufficient practical data.^38^, ^39^

Radiographic investigations of ultrasound, MRI, and X‐rays of the hand may show similar patterns like osteoarthritis or psoriatic arthritis features such as joint space narrowing, osteophytes, bone sclerosis of first carpometacarpal (CMC), MCP joints on both sides, and PIP joints.^40^, ^41^, ^42^ Also, central bone erosions, complete destruction of subchondral bone plate or typical gull‐wing or claw‐like hand deformity, posterior ribs notching.^33^, ^43^, ^44^, ^45^, ^46^

#### 4.2.2 Muscle involvement

Skeletal myopathy is the most frequently seen pathology in SSc patients, eventually affecting 5%−96% of the patients due to the lack of exact diagnosis specifications.^42^ A myriad number of antibodies are discovered that cause myositis, they are ACA, antibodies against anti‐Ku, PM/scl, anti‐U3‐RNP (fibrillarin) antibodies, anti‐RNAP III, anti‐RuvBL1/2 (pontin, reptin), anti‐scl70, anti‐PL12, anti‐PL7, anti‐SRP.^47^ Patients present with muscle fatigue, the diverse intensity of pain, symmetrical proximal or distal muscle weakness along with idiopathic inflammatory myositis: altogether restricts movement of muscle as sclerosis and fibrosis are formed in underlying tissues eventually necrosis of muscle, ultimately affecting the daily quality of life (QoL).^28^, ^48^ It further offenses cardiac and respiratory muscles leading to lethal diseases tend as myocarditis, left ventricle dysfunction, pericarditis, conduction defects, pulmonary fibrosis, decreased forced vital capacity (FVC), and PAH.^33^

The prognosis of SSc myositis is fatal if cardiac or lung involvement as they cause severe functional glitches as stated above, sans organ acquaintance is not problematic to patients.^42^ Unlike skeletal, myositis responds well to corticosteroids, glucocorticoids, methotrexate, rituximab, IVIG, and immunosuppressive therapy only for inflammation sans necrosis.^49^, ^50^, ^51^

#### 4.2.3 Tendon involvement

TFR, tenosynovitis, and tendon rupture are major pathologies; the prevalence rate is minimal, with just 11% in SSc; antibodies involved are anti‐topo I, anti‐centromere, anti‐scl70, anti‐RNAP III. The areas highly involved are ankles, anterior and posterior tibial, peroneal, and Achilles, followed by extensor or flexor tendons of fingers, knees, wrists, hand, elbows, triceps and shoulders, scapular, trochanteric, PIPs, MCPs, toe rubs.^31^, ^52^, ^53^, ^54^ TFRs are felted over the tendon along with pain complaints by patients, in addition, there will be movement restriction at joints due to tenosynovitis.

TFRs are reproducible, yet they can be infrequent or vanish with repeated movements, tenosynovitis includes an inflammatory environment around the tendon, which ultimately forms fibrosis and sclerosis, considering that tendon rupture is inevitable at the final stage.^42^

#### 4.2.4 Acro‐osteolysis

The resorption or erosion of distal phalanges makes them shortening more likely to occur in bones of hands and feet, anti‐scl‐70, ANA.^55^ It has a prevalence of approximately 6%−65% in SSc patients. Chronic hypoxia plays a pivotal role in bone resorption by osteoclastogenesis, osteoclasts, osteolysis, or angiogenesis. The main pathway behind this is the (hypoxia‐inducible factor [HIF‐1∝]/VEGF) signaling mechanism in regulating osteoclastic bone resorption as well as angiogenesis.^56^ Following this, there is a rapid increase in receptor activator of nuclear factor kappa beta ligand (RANK‐L) (osteoblasts precursor) and monocyte colony‐stimulating factor (M‐CSF), which actively participate in the pathophysiology of the SSc.^57^ It is associated with DUs, extra‐articular calcification,^58^ ILD, reduced FVC, PAH,^57^ Raynaud phenomenon, myocardial fibrosis, cardiac effusion, arthralgia, myositis, erectile dysfunction, arterial hypertension, erosive esophagitis, gastroesophageal reflux disease.^55^, ^59^, ^60^

#### 4.2.5 Calcinosis

Similar damage is engraved in both lcSSc and dsSSc patients, the prevalence is around 18%−38%^61^ and ACA, anti‐PM/Scl‐70 are evident.^62^ It is associated with calcium hydroxyapatite crystal deposition in subcutaneous tissues in both diffuse and limited SSc.^27^ The erosions are evident anywhere in telangiectasis, digital ischemia, sclerodactyly, nail‐fold capillary abnormalities, DUs and effects hands, wrist, gluteal, iliac crests, spine,^31^ forearms, elbows, DIPS, MCPS, fingertips.^63^ CT, X‐ray, PET‐CT, power Doppler, and MRI are used to identify calcinosis,^61^ multidetector computed tomography, and dual‐energy computed tomography 3D CT.^46^ Calcinosis is so vulnerable to infection or ulceration; strict monitoring is warranted. A classification system described it as visible and palpable, non‐visible and palpable.^64^, ^65^, ^66^, ^67^, ^68^

### 4.3 Renal involvement in scleroderma

**Figure 2:**
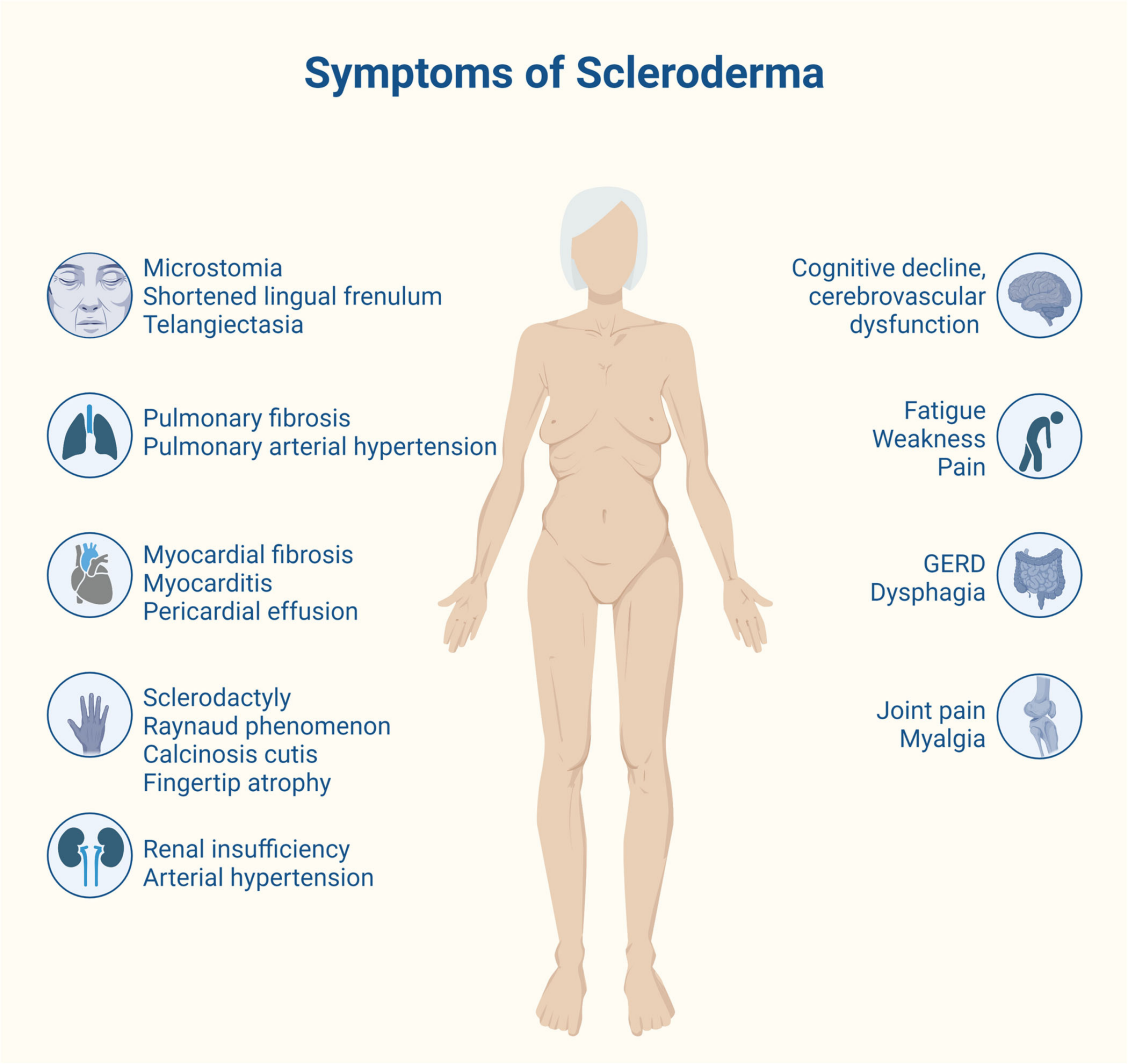
A summary of the signs and symptoms seen in scleroderma patients (original figure, made with Biorender).

SSc could lead to acute or chronic renal problems. Among them, renal crisis is the riskiest condition (Figure 2). The prevalence is high in dcSSc, with 10% when compared to lcSSc is very rare, with just 0.5%^42^ and 2.4% in the entire SSc patients.^69^ In contrast to past scenarios, currently hypertension, cardiac, lung, renal disease, and atherosclerosis are now accepted as primary involvement of SSc.^33^, ^70^ The risk factors are glucocorticoids, prednisone (>15 mg), corticosteroids, cyclosporine therapy, early age <4 years,^25^, ^30^ rapidly progressing skin disease or TFRs, presence of antibodies,^71^ robust association between drugs which cause vasoconstriction are tacrolimus, cyclophosphamide, cocaine.^42^ In the ancient days, it was a dreadful pathological condition for SSc patients. Nevertheless, angiotensin‐converting enzyme (ACE) inhibitors revolutionized the outcomes and reduced renal complications, making it a treatable disease.^25^, ^33^, ^72^ Antibody formation against RNA polymerase 3, anti‐topoisomerase I, anti‐U3RNP, and anti‐Th/To^23^, ^73^ increments the risk of a renal crisis in dcSSc patients.^74^, ^75^ The overall 5 year‐mortality rate of scleroderma renal crisis (SRC) patients out of specialized centers is high ranging from 30% to 50%.^13^

#### 4.3.1 Pathogenesis of renal damage

The basic plot of disease inflammation, fibrosis, and vascular injury similar patterns is in vascular injury, this is most awful as well as a paramount vital event in SSc pathogenesis.^76^, ^77^ The pathophysiology is not yet known lucidly, albeit various researchers proposed certain mechanisms as vasculature is the prime goal and early event.^76^, ^78^ Few antibodies are considered to be taking part in vascular destruction, firstly anti‐endothelial cell antibodies target topo‐isomerase I antigen. Other antibodies are anticentromere, anti‐topo‐isomerase I, anti‐scl‐70, antibodies against angiotensin II type 1 receptor, antiangiogenic receptor CD36, endothelin‐1 (ET‐1) type A receptor, anti‐annexin V receptors are involved.^79^, ^80^ Microvascular injuries cause proliferative vasculopathy, which causes an increase or overprovision of endothelial cells and smooth muscle cells, which further causes intimal thickening and occlusion.^81^ Endothelial cells get damaged, which is associated with the production of endothelin‐1 (ET‐1), von‐Willebrand factor (vWF), low levels of nitric oxide (NO), and endothelin NO synthase.^82^

This imbalance of blood flow results in alternate vasodilation and vasoconstriction and attracts macrophages or mononuclear cell infiltration, and perivascular inflammatory cell infiltrates.^83^ ET‐1 plays a crucial part in the differentiation of fibroblasts into myofibroblastic phenotype, which in turn increases the chances of the thickening of the inner layer of blood vessels and the narrowing of the vessels of the juxtaglomerular apparatus.^84^, ^85^ Following this, arteries and capillaries lose their elasticity, media, and adventitia, turning fibrotic.^70^ Proangiogenic and antiangiogenic factors have an imperative role in further disease events. Despite proangiogenic factors such as IL‐8, VEGF, and basic fibroblast growth factor boost angiogenesis (Table 1); ultimately, angiogenesis is dysregulated by unknown process.^86^, ^87^

**Table 1:** Proangiogenic, antiangiogenic factors and their respective molecules involved in the pathophysiology of renal insult in scleroderma.

Type of factor	Molecule
Proangiogenic	Vascular endothelial growth factor (VEGF)
	Vascular cell adhesion molecule‐1 (VCAM‐1)
	Transforming growth factor beta (TGF‐β) and endoglin (ENG), platelet‐derived growth factor (PDGF)
	Stromal cell‐derived factor 1 (SDF‐1/CXCL12)
	P/E‐selectin
	Basic fibroblast, platelet‐derived growth factors
	CD44
	Interleukin (IL)‐8/CXCL8
	uPAR and kallikreins 9, 11, and 12
	Adhesion molecules
	Hepatocyte growth factor (HGF)
	Platelet‐activating growth factor (PAF)
	Tumor necrosis factor‐α (TNF‐α)
	Kallikrein 11 &12
	Tissue inhibitors of metalloproteinases
	Monocyte chemoattractant protein‐1 (MCP‐1)
	IL‐6
	β‐thromboglobulin
	Tumor necrosis factor‐α (TNF‐α)
	Endothelin‐1 (ET‐1)
	Urokinase plasminogen activator receptor (uPAR)
	Endostatin
	Angiostatin
	Kallikrein 3
	Platelet factor 4 (PF4)
	Thrombospondin‐1 (TSP‐1)
	TSP‐2
	Monokine induced by interferon‐γ (MIG/CXCL9)
Antiangiogenic	Interferon inducible protein‐10 (IP‐10/CXCL10) IL‐4 & 12
	Pentraxin 3 (PTX3)
	Thrombospondin‐1
	Angiopoietin‐2
	Tissue inhibitors of metalloproteinases
	Pentraxin‐3 (PTX3)
	Interferon‐α and ‐γ

Not only this normal function of bone marrow‐derived endothelial progenitor cells (EPCs) is altered, which is essential for vasculogenesis. Based on the evidence, we can conclude that there is an alteration or impairment in the vasculogenesis mediated by EPCs.^70^, ^78^, ^88^ Soluble adhesion molecules such as intracellular adhesion molecule 1 (ICAM‐1), endothelial leukocyte adhesion molecule 1 (ECAM‐1), vascular adhesion molecule 1 (VCAM‐1), E‐selectin, P‐selectin in ECs, these are proangiogenic markers,^89^, ^90^ cytokines, chemokines. Disease progression grounds loss of microvasculature in major organs, reduced capillaries make oxygen and nutrient supply arduous to the organs, and ultimately hypoxia prevails, it surges the production of HIF. HIF triggers fibrosis and an increase in VEGF. From then, hypoxia provokes the need for vasculogenesis and angiogenesis, to make situations worse vascular recovery is altered significantly, and avascular regions are prominent.^91^ Bone marrow‐derived EPCs participate in the formation and reconstruction of the damaged vessel; they are recognized by certain biomarkers like CD 133, CD34, and vascular endothelial growth factor receptor 2 (VEGFR 2)^79^: another subset of EPCs are monocytic EPCs with biomarker CD14, and they possess the capability to differentiate into endothelial capillary cells (ECs), pericytes as well as smooth muscle cells.^88^, ^92^, ^93^

Many research studies produced varied results some stated there is drastic raise in EPCs although a few acclaimed minimal amounts of EPCs present and others elucidated that EPCs have functional impairment in SSc individuals.^88^, ^94^ Nonetheless, on the whole, Del Papa et al. confirmed that EPC‐induced angiogenesis is disappeared in SSc patients.^92^ Angiogenesis is always promoted by proangiogenic mediators, which are called proteolytic enzymes, which include matrix metalloproteinases. It erodes the EC basement membrane and perivascular matrix.^94^, ^95^, ^96^, ^97^, ^98^, ^99^

Subsequently, some angiogenic mediators stimulate ECs to proliferate and migrate toward degraded areas after matrix degradation. Therefore, ECs form new sprouts, which then develop into mesenchymal cell recruitment, capillary formations, and basement membrane formation.^100^, ^101^, ^102^ In SSc patients, proangiogenesis factors are well exceeded than antiangiogenesis (Table 1) for all factors.^100^, ^103^, ^104^

#### 4.3.2 Characteristics of renal crisis

Various presentations of kidney pathology in SSc are hypertensive SRC, normotensive SRC, isolated reduced glomerular filtration rate, myeloperoxidase anti‐neutrophil cytoplasmic antibody‐glomerulonephritis (MPO‐ANCA GN), reduced renal function reserve and disparate renal vascular resistance indices.^104^ The basic pathway of renal crisis is thrombotic micro‐angiopathy (hemolytic anemia), acute pulmonary edema, malignant hypertension >150/90 mmHg (hyperreninemia), and progressive acute kidney injury,^30^, ^105^ 50% of patients have worsening renal function.^51^ Biopsy findings indicate the renal plasma flow measurements that vascular intimal proliferation leads to typical onion bulb‐like lesions characterized by vessel narrowing and resting renal blood flow (RBF).^84^, ^106^, ^107^

These changes decrease renal perfusion, precisely RBF eventually renal ischemia.^107^, ^108^ The renin‐angiotensin system is responsible for vascular alterations after vascular endothelial damage: it tries to stabilize blood pressure and maintains hydro‐mineral homeostasis. A sharp rise in renin levels and angiotensin II are linked to the onset of malignant hypertension and renal failure is discovered, and the mechanism is yet unknown.^107^ It has a peculiar presentation with rapid onset hypertension, headache, fatigue, dyspnea, hypertensive retinopathy, and acute left ventricular failure along with retention of nitrogen bodies because of acute progressive renal failure.^42^, ^75^, ^109^, ^110^ Other symptoms are headache, blurred vision, shortness of breath, seldom encephalopathy, and seizures. Oliguric renal failure may cause congestive heart failure, arrhythmias, pericarditis or pericardial effusions, myocardial fibrosis, increased peripheral vascular resistance contributes to PAH or heart failure, myocardial dysfunction, cardiac arrest, PAH, thrombocytopenic purpura.^105^

US and Doppler US are the gold standard noninvasive tests for analyzing the kidney as well as the urinary tract. Especially Doppler US is used for measuring renal resistive index, pulsative index, and systolic‐diastolic blood flow.^108^, ^111^ It is so beneficial in assessing parenchymal fibrosis, renal vascular resistances or compliance studies, arterial hypertension and sclerosis, and inflammatory changes.^106^ The other methods are angiography and renal scintigraphy (99m‐technetium‐diethylnitriaminepenta‐acetic acid [99m‐Tc‐DTPA]).^104^

ACE inhibitors (ACEi) captopril, and enalapril are the mainstay for treating SRC. There is authentic evidence in the usage of angiotensin II receptor blockers (ARB II) and glucocorticoids, so, care must be taken while giving them.^112^, ^113^ BP monitoring is done every three times per week in severe patients for others it's just once or twice per week.^71^ Other anti‐hypertensives are dihydropyridines (CCBs), diuretics, alpha‐blockers and beta‐blockers,^113^ Endothelin‐1 antagonists bosentan, plasma exchange, and Ecolizumab.^74^, ^114^ Kidney transplantation (KT) is an ideal option for SSc patients who are not recovering through dialysis procedures in the time span of 1 year. It also gives them relief from ample symptoms and provides a QoL.^115^ Albeit, the outcomes of KT are worse in SSc, the 5‐year patient survival and graft survival, and 3‐year graft survival are 74.7%, 56.7%, and 60.3%, respectively.^109^

Immunosuppressive agents preferred in SSc are cyclosporine, corticosteroids, anti‐lymphocyte serum, anti‐interleukin 2 receptor, tacrolimus, mycophenolate mofetil, calcineurin inhibitors (CNIs), belatacept or mammalian target of rapamycin (mTOR) inhibitors, each drug has its own side effects exceptionally in SSc because of the severity of the disease itself. Due to discrepancies, some opt to choose to treat on an individual basis, while some follow specific regimes of mTOR inhibitors, mycophenolate mofetil, and CNIs.^110^, ^115^ Irrespective of the stage or relapse of SRC, RT patients should receive ACEis due to their potential hypertension conditions. Even though a recent study acclaimed ARB 2s are beneficial who switched from ACEis, still many no significant evidence to support it exactly.^115^

### 4.4 Cardiac involvement in scleroderma

#### 4.4.1 Conduction defects and tachyarrhythmias

Estimates of conduction defects in SSc range from 4% to 51%, depending on whether resting electrocardiogram (ECG) or 24 h ambulatory ECG monitoring is used.^116^ Conduction system disease, such as bundle branch blocks and/or atrioventricular blocks, is likely due to fibrosis of the conduction system from recurrent microvascular ischemic insult and autonomic dysfunction.^117^, ^118^ Symptomatic supraventricular tachyarrhythmias tend to be more common than bradyarrhythmias.^119^, ^120^, ^121^ Ventricular arrhythmias are less common but are associated with an increased risk of sudden death and mortality, especially when concurrent with skeletal myopathies and systolic dysfunction.^122^, ^123^

#### 4.4.2 Autonomic insufficiency

Autonomic insufficiency is frequent in SSc, occurs at the early stages of the disease process, and might precede the development of myocardial fibrosis.^124^, ^125^ Lack of heart rate variability and resting tachycardia are predictive of increased mortality in SSc.^53^ Patients with SSc and autonomic insufficiency present similarly to that of the general population, with symptoms of positional dizziness or orthostatic hypotension, inappropriate heart rate response to exertion or exercise intolerance, and inappropriate sweating or sensation of warmth. In some cases, these symptoms may be associated with reflex syncope.

#### 4.4.3 Heart failure

Many asymptomatic patients with dcSSc who undergo CMR are found to have myocardial inflammation and/or diminished myocardial perfusion reserve index with evidence of diffuse fibrosis despite normal routine cardiac evaluation.^126^, ^127^, ^128^ These findings suggest that early myocardial involvement in SSc can occur unaccompanied by cardiac symptoms. Clinically active myocarditis, while uncommon, is associated with substantial morbidity and mortality and is typically associated with myositis and overlap syndromes.

### 4.5 Neurological involvement in scleroderma

Neurological involvement in scleroderma is usually considered rare and secondary to the disease process, but recent times have suggested otherwise and led to consideration of nervous system involvement as a primary pathologic process in scleroderma.^129^ It can cause damage to the peripheral nervous system, leading to neuropathy which can cause sensory and motor disturbances. Central nervous system effects can present in the form of Schizophrenia like symptoms and paranoid hallucinatory syndromes.^130^ Seizures and white matter lesions have also been reported in linear scleroderma en coupe de sabre (LSES) and parry Romberg syndrome variants of localized scleroderma.^131^

Pathophysiology behind these effects is less understood, however recent developments have shown that SSc may cause a significant primary vascular change in the brain involving advanced calcification in arterioles and capillaries of brain parenchyma. This change in vasculature is found in almost every organ of patients affected with SSc.^132^ Involvement of endothelial cell damage in brain parenchyma is thought to be main reason behind these effects that is triggered by autoantibodies, reactive oxygen species which may ultimately lead to luminal narrowing, vessel obliteration and intimal hyperplasia.^133^ This articulates with the fact that increased levels of cell adhesion molecules also have been shown to correlate with increased disease severity and ultimately lead to chronic inflammatory changes in CNS vasculature.^134^, ^135^

Compression of nerve fibers as a result of the increased thickening of perineurium and endoneurium can also be the main underlying process and have been shown to cause symptoms in peripheral nerves in the form of entrapment syndromes involving cases of Carpal Tunnel Syndrome, which showed resistance to treatment through conventional surgical methods thus, were thought to be due to underlying diffuse damage to peripheral nervous system.^136^ Moreover, there has been significant evidence of cognitive decline and cerebrovascular dysfunction in patients affected with scleroderma and has been linked due to problems in adequate cerebral perfusion, which most probably occurs due to damage to vasculature especially involving endothelial cell damage as discussed above,^137^ however more research is needed to explore the complex mechanisms that cause changes in central and peripheral nervous system.

### 4.6 Scleroderma genetics

The symptoms of scleroderma are vague, affecting different body areas in various ways. As a result, scleroderma diagnosis might be challenging. Most of the time, the procedure starts with a complete physical examination during which the doctor will look for any changes in your skin that may be indicators of the ailment. The physician can next advise a blood test to look for high antibody levels. A proper diagnosis requires a blood test because this is a sign of an autoimmune illness.

The illness could also be confirmed by other diagnostic testing. This may entail skin biopsies, a urinalysis, a CT scan of the lungs, and chest X‐rays.^138^ The main strategy for keeping track of scleroderma is careful clinical examination. To check for anomalies, X‐rays and CT scans are performed. Thermography can identify skin temperature variations between a lesion and healthy tissue. Ultrasound and MRI might be of use in soft tissue assessment.^139^

ANAs, which are immunological factors, can be found by serology or antibody testing. Diagnosing scleroderma can be aided by identifying certain ANA subtypes.^139^ Autoantibodies, such as those against RF, single‐stranded DNA, and histones, are connected to scleroderma, but they are also widespread in autoimmune diseases such as RA and systemic lupus erythematosus. Some ANAs target the genetic material in cells, such as RNA or DNA. Autoantibodies are linked to scleroderma, but other autoimmune disorders, such as systemic lupus erythematosus and RA, can present with them too. These autoantibodies comprise those that are directed against histones, single‐stranded DNA, and RF, as examples.^138^, ^140^ Certain ANAs concentrate on a type of RNA or DNA present in the cell's genetic component. Anti‐RNA polymerase III, anti‐topoisomerase I (TOPO, also known as anti‐DNA topo 1), and ACA are further types of auto anti‐bodies. One or more of these autoantibodies are usually present in patients with SSc, but this is not the case with localized scleroderma.

### 4.7 Therapeutic updates

Drug trials have been started due to understanding and mediators in systemic scleroderma. Janus kinase (JAK), lysophosphatic acid receptor 1 (LPA 1 receptor), IL‐6, tumor necrosis factor, autotaxin, CD28‐CD80/86, CCL24, CD30, CD‐19, CD‐20, TGF soluble guanylate cyclase (sGC), B‐cell activating factor (BAFF), and endothelin receptor have been identified as the therapeutic targets. Data from clinical trials of these drugs indicate a high potential for a variety of innovative SSc therapy alternatives in the coming years.^141^

To optimize the chance of detecting the probable causal variants, gene identification studies—which extended further than the initial wave of GWAS investigations—have subsequently attempted to collect as much genetic variation as feasible in each region. Resequencing, Fine mapping, and imputation of variations not genotyped directly are some methods used. Numerous publications introduced possible candidate genes in separate cohorts since the first GWAS and, more recently, Immunochip investigations, often with statistical significance below suggestive evidence for connection. Therefore, it is important to evaluate association data with care.^142^

The initial Immunochip study's^143^ findings, which showed significant SSc correlations in the DNASE1L3‐PXK, IL12A, ATG5, and TREH‐DDX6 areas, were published at the beginning of 2014. At the same time, a GWAS further investigation^144^ discovered a potential link to PPARG. Later that year, the relationship in the DNASE1L3‐PXK area was verified by a second Immunochip research, which revealed a novel, suggestive link with VCAM1.^8^ More recently, a candidate gene research^145^, ^146^ found intriguing evidence of a link between PLCL2 and SSc, and an Immunochip follow‐up study found a substantial correlation with IL12RB1.^147^

The causative variations are yet unknown, as is the case with most documented connections. The signal most likely generated by a missense mutation in DNASE1L3 is only that in the DNASE1L3‐PXK region. This correlation is higher in both ACA‐positive and lcSSc instances, which is consistent with the relationship between ACA antibody status and lcSSc. Given that DNASE1L3 participates in DNA degradation during apoptosis, the protein's loss of function and ensuing abnormalities in DNA clearance may be related to the generation of ACA.^143^, ^145^

It is not unexpected that most of the involved genes are involved in immunological processes, given that the Immunochip array targets immune‐related genes. ATG5 plays a part in autophagy, and both IL12A and ATG5 are engaged in interferon signaling. A part of the IL12 pathway is IL12RB1. VCAM1 mediates leukocyte‐endothelial cell attachment and signaling. B‐cell proliferation and B‐cell receptor signaling are both facilitated by PLCL2. However, one of these recently discovered genes, PPARG, is an antifibrotic factor that could impact the uninhibited development of fibrosis in SSc.^144^ These correlations support the hypothesis that abnormalities in DNA degeneration, interferon signaling, autophagy, IL12 signaling, B‐cell signaling, cell adhesion, and fibrotic processes contribute to this disorder's pathology.

Although fibroblast activation is unquestionably a feature of SSc, most of the genetic variables connected to SSc are immune‐related genes^148^, ^149^ and the causes of the SSc's distinctive excess deposition of ECM proteins are still unknown. In contrast to idiopathic pulmonary fibrosis, SSc‐associated ILD^150^ has a distinctive genetic architecture of pulmonary fibrosis that is noteworthy. This suggests that these two types of lung fibrosis have different genetic risks and that the SSc‐associated ILD may have a more immune‐driven cause of fibrosis. It was recently confirmed that the genetic risk in SSc was connected mainly to immunological problems by a bioinformatic study of gene expression data from skin biopsies combined with SSc‐associated genetic variation. Their findings provide more credence to the idea that immune system stimulation is a crucial and early stage in the development of SSc, possibly involving the activation of interferon and the migration of macrophages, that might affect or promote ECM remodeling and skin cell multiplication^151^ Trials in SSc with possible positive, negative, and open enrollment.^152^

#### 4.7.1 Bosentan

Bosentan is the first orally active agent to be approved for treating pulmonary hypertension with high‐affinity endothelin dual receptor antagonism. As discussed previously, SSc is characterized by rising levels in plasma and enhanced expression of endothelin‐1 in tissues, and patients exhibit overexpression of the endothelin receptor in the affected organs and tissues.^153^, ^154^, ^155^, ^156^ In two randomized studies, Bosentan has been shown to be beneficial in treating both PAH caused by connective tissue disorders and idiopathic PAH.^157^, ^158^

#### 4.7.2 Halofuginone

In both scleroderma and chronic graft‐versus‐host disease (cGvHD), halofuginone has been utilized to treat fibrosis.^155^, ^156^ Clinical traits, such as skin and internal organs fibrosis, are shared by SSc and cGvHD. Regardless of the underlying reason, ECM deposition—mainly consisting of collagen type I—defines fibrosis: internal organ failure and the increasing buildup of connective tissue lead to the breakdown of normal tissue architecture. The degree of skin and internal organ fibrosis corresponds with the progress of the illness in both SSc and cGvHD.^159^

#### 4.7.3 Infliximab

Infliximab 5 mg/kg was administered to patients at weeks 0, 2, 6, 14, and 22. Utilizing the physician global assessment on the visual analog scale, modified Rodnan skin score (mRSS), and the SSc functional score, the primary effectiveness aim was to evaluate the progress from baseline to Week 26. Evaluation of infliximab's safety and acceptability in dcSSc was a major secondary goal of the trial. Although a substantial effect at 6 months was not seen, infliximab appears to stabilize cutaneous diseases and may temporarily relieve skin sclerosis.^155^, ^156^

#### 4.7.4 Ximedon

A pyrimidine chemical, ximedon, was administered during electrophoresis to the afflicted skin and limbs in a double‐blind, placebo‐controlled experiment on 56 patients with SSc. SSc patients' conditions were improved in 77.8% of cases, microhemo‐circulation was improved in 72.2% of cases, and the afflicted skin area was reduced by 9.8% (*p* 0.05), resulting in skin induration in 55.6% of cases.^160^

#### 4.7.5 Photopheresis

A randomized, placebo‐controlled, double‐blind study on photopheresis in SSc revealed that it significantly reduced skin and joint complications in recently diagnosed scleroderma patients. A potential placebo effect may be minimal.^161^

#### 4.7.6 Cyclophosphamide

According to an experiment conducted with placebo‐control, 1 year of oral cyclophosphamide treatment had a modestly positive impact on the function of the lungs, dyspnea, skin thickness, and overall QoL in scleroderma‐induced ILD. Throughout the entire research period, the impacts on lung function persisted.^162^

#### 4.7.7 Quinapril

Quinapril in sclerosis, which can potentially replace the current ACEi treatments in PSS, is one of the medications included in the multicentric trial known as the QUINS trial, which was recently launched.^160^

#### 4.7.8 Clinical trial scleroderma‐cyclophosphamide and transplantation (SCOT)

SCOT is the name of an ongoing experiment that will contrast two possible treatments: high‐dose cyclophosphamide administered monthly and transplantation of autologous stem cells.^163^

#### 4.7.9 Penicillamine

A definitive trial on this medication contrasted low‐dose 120 mg on alternate days with 822 mg daily.^164^ According to the study, the difference between the two doses was not statistically significant, and the medicine was no more effective than a placebo. Recent research^165^ randomly chose 84 individuals in retrospect who had diffuse epidermal SSc and had been administered d‐penicillamine around 24 months after the clinically apparent start of skin sclerosis, is now raising interest. Skin, heart, pulmonary, and renal involvement showed statistically significant improvement at a median dosage of 750 mg daily. The study concluded that d‐penicillamine therapy at an average dose of 750 mg per day can significantly reduce skin participation and improve renal, heart, and pulmonary functioning in people via dcSSc, a progressive disease with new onset.

The drugs and their mechanism of action are summarized in Table 2.

**Table 2:** Drug trials and their mechanism of action in scleroderma.

Drug	Mechanism of action
Bosentan	Endothelin dual receptor antagonism
Halofuginone	Inhibition of fibrosis progression
Infliximab	Tumor necrosis factor inhibition
Ximedon	Pyrimidine chemical administration
Photopheresis	Immunomodulatory effects
Cyclophosphamide	Immunosuppressive and antifibrotic effects
Quinapril	Angiotensin‐converting enzyme inhibitor
SCOT trial	High‐dose cyclophosphamide versus autologous stem cell transplantation
Penicillamine	Metal chelation and immunomodulation

Abbreviation: SCOT, scleroderma‐cyclophosphamide and transplantation.

### 4.8 Limitations

As with any review, our study had a fair few limitation points. While we made efforts to conduct a thorough search across multiple databases, the exclusion of studies published in other languages might limit the comprehensiveness of our review. The heterogeneity of studies on scleroderma is another important consideration. As different studies carried different variations in patient populations, disease severity, treatment protocols, and study designs, this may hinder direct comparisons and synthesis of results. Our review may also identify certain research gaps or areas with limited investigations in scleroderma. These gaps should be acknowledged as potential opportunities for future research to address remaining knowledge gaps and drive advancements in the field.

## 5 CONCLUSION

The review provides a comprehensive overview of scleroderma, an intricate autoimmune disease distinguished by fibrosis and vascular abnormalities. The pathological processes involved in scleroderma contribute to a wide array of clinical manifestations, affecting multiple organ systems. Accurate diagnosis, which involves clinical evaluation, serological testing, and imaging studies, is crucial for appropriate management. Treatment strategies aim to alleviate symptoms, manage complications, and slow disease progression, necessitating a multidisciplinary approach.

## AUTHOR CONTRIBUTIONS

**Priyadarshi Prajjwal**: Conceptualization; methodology; validation; writing—original draft. **Mohammed Dheyaa Marsool Marsool**: Methodology; resources; validation; writing—original draft; writing—review and editing. **Vikas Yadav**: Validation; writing—original draft. **Ramya S. D. Kanagala**: Writing—original draft; writing—review and editing. **Yeruva Bheemeswara Reddy**: Resources; supervision; validation; writing—original draft. **Jobby John**: Writing—original draft; writing—review and editing. **Justin Riley Lam**: Visualization; writing—review and editing. **Nanditha Karra**: Visualization; writing—original draft. **Bita Amiri**: Visualization; writing—original draft. **Moiz Ul Islam**: Visualization; writing—original draft. **Venkatesh Nithya**: Writing—original draft; writing—review and editing. **Ali Dheyaa Marsool Marsool**: Visualization; writing—original draft. **Srikanth Gadam**: Writing—review and editing. **Neel Vora**: Writing—review and editing. **Omniat Amir Hussin**: Writing—review and editing.

## CONFLICT OF INTEREST STATEMENT

The authors declare no conflict of interest.

## TRANSPARENCY STATEMENT

The lead author, Omniat Amir Hussin, affirms that this manuscript is an honest, accurate, and transparent account of the study being reported, that no important aspects of the study have been omitted, and that any discrepancies from the study as planned (and, if relevant, registered) have been explained.

## Data Availability

All the data used in this study are present within the study itself. No new data were created or analyzed in this study.
